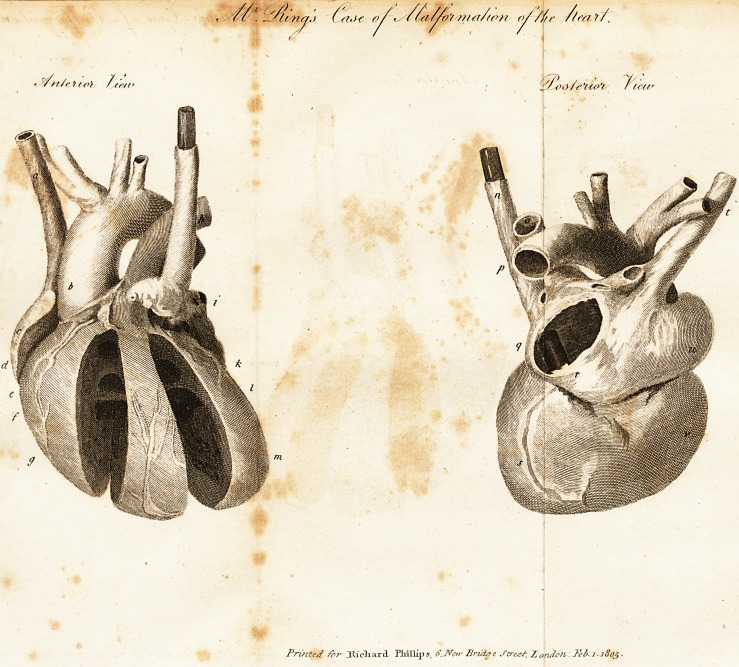# A Singular Case of Malformation of the Heart

**Published:** 1805-02-01

**Authors:** 


### A singular Case of Malformation of the Heart; *Communicated by* Mr. Ring.

[ With an Engraving. ]

An account of appearances observed, in examining the bodies of those who die of incurable diseases, is of no small importance. It enables the practitioner to form a just prognostic in similar cases; prevents the patient from being tormented with painful and useless applications; and furnishes the physiologist with means of ascertaining the functions of the various parts of the animal economy, which could not have been so well ascertained from an examination of those parts in a healthy state. Hence it may be useful to insert in your Journal, the following history of a singular deviation from the natural structure of the heart.

Caroline Mergauts, No. 152, Swallow Street, was born June 20, 1802. From the time of her birth, she laboured under a great difficulty of breathing. Her complexion was, in some parts, of a lead colour; in others, of a bluish purple; particularly in the lips.

Twice every day she had fits of crying, or rather screaming, which lasted several hours; during which the chest was much convulsed.

The paroxysms gradually became longer, and more violent; so that, at length, she had scarcely an hour's interval of ease either day or night. She suffered, in consequence, the most extraordinary degree of emaciation. During the last fortnight of her life, she seemed to take no notice of any thing. She died on the 20th of June 1803, which was the first anniversary of her birth.

I was consulted, when she was five months old, concerning this case; which had been mistaken for one of common convulsions. I communicated to the mother my opinion of the nature of the disorder; and endeavoured, by anodynes and antispasmodics, to alleviate those sufferings which the healing art could not cure.

After the death of the child, leave having been obtained to examine the body, I requested Mr. Astley Cooper to undertake that office; Dr. Jenner, Dr. J. Walker, and myself, being present.

### *Appearances after Death.*

The heart was the only viscus, in which there appeared any remarkable deviation from the natural structure. It was thicker than common at the apex; approaching rather to the form of a cube, than of a cone.

The

### M^r. Ring's Case of Malformation of the heart.

Anterior View

Posterior View

Printed for Richard Phillips, 6, New Bridge Street, London. Feb. 1. 1805.

The ventricles bore a greater resemblance to those of the turtle, than of the human heart. There was a large opening in the septum of the ventricles, near the aorta. The septum of the auricles was totally wanting.

The vessels were connected with this organ in the following manner. The inferior cava entered its basis on the left side instead of the right, passing through the centre of the diaphragm ; and the fissure for that purpose, in the liver, was more on the left side than usual.

There were two superior cavæ ; one terminating in the usual manner, in the right auricle, and the other in the left ; if they could properly be distinguished into right and left, when there was no septum.

The aorta arose from both ventricles ; not, as usual, from the left alone. The pulmonary artery and veins pursued their ordinary course ; but the pulmonary artery was much smaller than usual.

Deviations from the natural structure of the heart, producing blueness in the skin, such as existed in this case, are not unfrequent ; but in the present instance, the deviation was one of the greatest that has hitherto been described. In a common case of malformation of the heart, in which the aorta arises from both ventricles, a deficiency in the septum of the ventricles has also been found, at the part where the aorta arises. Hence it must receive venous blood, as well as arterial : yet, as that part of the vessel which arrives from the right ventricle is smaller than the other, it receives less venous than arterial blood. Such a defect in the vascular system, and the defect of oxygenation, which is the necessary consequence of a want of due circulation through the lungs, is apparent in every part of the body ; particularly in the most vascular parts, such as the lips and cheeks ; which, instead of a florid colour, are of a livid hue.

But in the present instance, exclusive of those ordinary deviations from the common course of nature, a mixture of venous and arterial blood took place in the auricles, from the total want of a septum ; and would in some measure have taken place, had they been furnished with a septum, from two of the venæ cavæ terminating on the left side. The aorta, therefore, at both its origins, must have received partly venous, and partly arterial blood. Under all these difficulties, it is not surprising that this infant did not survive the period before mentioned. It is rather a wonder that she lived so long.

*New Street, Hanover Square.*

*Copy*

## Figures and Tables

**Figure f1:**